# The Predictive Effects of Gender and Academic Discipline on Foreign Language Enjoyment of Chinese High School Students

**DOI:** 10.3389/fpsyg.2021.802152

**Published:** 2022-01-04

**Authors:** Jian Huang, Guiying Jiang

**Affiliations:** ^1^School of Foreign Studies, Central University of Finance and Economics, Beijing, China; ^2^College of Foreign Languages and Cultures, Xiamen University, Xiamen, China

**Keywords:** foreign language enjoyment, gender, academic discipline, positive psychology, EFL learning

## Abstract

Foreign Language Enjoyment (FLE) plays a facilitative role in FL learning and its contributing factors have been the object of scholarly attention in the Positive Psychology approach to second language acquisition (SLA). The present study examined the predictive effects of gender and academic discipline on overall FLE and each of its subcomponents in a specific Chinese EFL context. Statistical analyses based on a sample of 1,718 high school students showed that: (1) female students scored significantly higher in overall FLE, *FLE-Private*, and *FLE-Atmosphere* than their male counterparts, but no significant difference was found in their *FLE-Teacher*; (2) students of Humanities and Social Sciences scored significantly higher in overall FLE, *FLE-Teacher* and *FLE-Atmosphere* than students of Natural Science, though no significant difference was identified for *FLE-Private*. Reasons for the statistical variations and their implications were also discussed.

## Introduction

Emotions are intimately involved in every aspect of learning process (Schutz and Lanehart, 2002) and in the field of SLA, researchers used to be predominately preoccupied with negative emotions such as anxiety (MacIntyre, 2017; Dewaele and Li, 2020). With the rise of the Positive Psychology movement, they began to explore positive emotions in the process of language learning. Foreign Language Enjoyment (FLE), one of the key positive emotions, is receiving increased scholarly attention for its role in promoting FL learning and learners’ mental well-being (Li, 2021). Levels of FLE have been found to be linked to many socio-biographical factors such as age, gender, education level, instruction level, and place of study (e.g., Dewaele and MacIntyre, 2014, 2016; Li et al., 2018). Dewaele and MacIntyre (2014) have found in their international corpus of 1,746 participants that female FL learners reported small but significantly higher levels of both FLE and Foreign Language Classroom Anxiety (FLCA) than male peers. An item-level analysis revealed that statistically significant differences were not found on all items (Dewaele et al., 2016). Female participants scored significantly higher on items referring to mild forms of FLCA such as worry and lack of confidence and -among the FLE items- they scored higher on fun, pride, excitement, enjoyment and interest in the FL. The authors were very careful in the interpretation of the findings, pointing out that researchers should not expect to find gender differences “on an inter-planetary level but rather on a detail-oriented, microscopic level” (Dewaele et al., 2016, p. 45). Dewaele and MacIntyre (2014) also found that the group of 229 Asian learners (of which 174 were Chinese university students) had the lowest levels of FLE and highest levels of FLCA compared to the European, Arab, South American and North American groups. This finding prompted Li et al. (2018) to investigate the uniqueness of FLE dimensions through exploratory and confirmatory factor analysis of data collected from more than 2,000 Chinese EFL learners using the *Chinese Version of Foreign Language Enjoyment Scale (CFLES).* The present study will investigate whether the gender patterns that emerged in Dewaele and MacIntyre (2014) and Dewaele et al. (2016) with a majority of multilingual Western participants with different FLs, will also appear in a unique Chinese high school EFL context characterized by unique learning and teaching styles (Xiao, 2006).

In Chinese high schools, the classes are most commonly divided into Humanities and Social Sciences (Politics, History, and Geography) and Natural Science (Physics, Chemistry, and Biology). English, together with Chinese and Mathematics, is obligatory for both classes as a FL with the same curriculum. The predictive effects of academic background or disciplines have been found on a variety of aspects in higher education (Krause, 2014), for example, on teaching/learning beliefs (e.g., Nevgi et al., 2004), teaching/learning approaches (e.g., Lindblom-Ylänne et al., 2006; Laird et al., 2008; Hill et al., 2016), dialogic behavior in course forums (e.g., Gorsky et al., 2010), teachers’ implicit requirements for their students (e.g., Neumann et al., 2002), students’ language use and discourse (Martin, 2011; Kuteeva and Airey, 2014), and students’ emotionality (Sánchez-Ruiz et al., 2010). The variables are inextricably intertwined with each other and affect the overall teaching and learning process. What remains largely unknown is whether the disciplinary differences identified in higher education also exist in secondary education. A final piece of the puzzle that merits further investigation is the role of FLE in Chinese EFL learning and variation linked to gender and academic discipline. Such research can lead to a better theoretical understanding of FLE by identifying its unique features and sources of variation in the Chinese context (Li, 2021).

## Literature Review

### Foreign Language Enjoyment

Influenced by Positive Psychology, positive emotions have attracted increasing scholarly attention in the field of SLA, especially because of their role in facilitating FL learning and promoting personal well-being (MacIntyre et al., 2016; Dewaele and Li, 2020; Li, 2021; Wang et al., 2021). Foreign Language Enjoyment (FLE), like other positive emotions, has been situated within the emerging field and theoretically grounded on the Broaden-and-Build Theory of positive emotions (Fredrickson, 2001, 2013) and the Control-Value Theory of achievement emotions (Pekrun, 2006; Pekrun et al., 2007). According to the Broaden-and-Build Theory, FLE can enhance FL learning in that it shares with other positive emotions the ability to “broaden people’s momentary thought-action repertoires, which in turn serve to build their enduring personal resources, ranging from physical and intellectual resources to social and psychological resources” (Fredrickson, 2001, p. 218). According to the Control-value Theory, enjoyment can also facilitate FL learning in that “experiencing enjoyment while working on a challenging project can help a student envision goals, promote creative and flexible problem solving, and support self-regulation” (Pekrun and Linnenbrink-Garcia, 2014, p. 4). Taken together, in FL learning, ‘enjoyment may be the emotional key to unlocking FL learners’ learning potential’ (Dewaele and MacIntyre, 2014, p. 261), because it can “enhance the ability to notice things in the environment, enhance awareness of language input, and dissipate the lingering effects of negative arousal, promote personal resiliency and hardiness during tough times” (Dewaele et al., 2016, p. 46).

The exploration of FLE initiated by Dewaele and MacIntyre (2014) focused on three strands: The first one dealt with the conceptualization and measurement of FLE. Using a large international sample, Dewaele and MacIntyre (2014) explored the relationship between FLE and FLCA, which turned out to be independent dimensions. Dewaele and MacIntyre (2016) found that a Principal Component Analysis of the 21 FLE items yielded two dimensions: *FLE-Social* (“positive feelings boosted by encouraging peers, nice teachers and a supportive environment” (p. 225) and *FLE-Private* (‘thoughts and feelings coalesce around a sense of accomplishment’ (p. 228). The FLE scale was later reduced to 10 items, retaining the two FLE dimensions (Dewaele and Dewaele, 2017; Dewaele and Alfawzan, 2018). The second (parallel) strand focused on qualitative data to complement the statistical analyses. Participants’ feedback on an open question enquiring about their enjoyable episodes in the FL class was coded. Forty-one percent of the comments were related to specific classroom activities, 20% were linked to the teacher and 14% were instances of peer recognition (Dewaele and MacIntyre, 2014, p. 256).

The final parallel strand dealt with the potential links between FLE and FL learning and a range of learner-internal, learner-external and teacher-centered variables involved in FL learning including age, gender, place of study, education level, instruction level, number of languages acquired, FL being studied, FL mastery, relative standing among peers, attitude toward FL, attitude toward FL teachers, teacher predictability, frequency of teachers’ use of FL, proportion of time spent on speaking, and FLCA (e.g., Dewaele and MacIntyre, 2014, 2016; Dewaele and Dewaele, 2017; Boudreau et al., 2018; Resnik and Dewaele, 2019; Guo, 2021). Li et al. (2018) initiated FLE research in China with an investigation of Chinese high school students’ conceptualization of FLE. Three factors emerged from an 11-item *Chinese Version of FLE Scale (CFLES)* based on Dewaele and MacIntyre (2016): *FLE-Private, FLE-Teacher*, and *FLE-Atmosphere*. The three factors revealed in this study focused on the context of China are slightly different from what was identified in counterpart studies conducted in different contexts, implying the need to adopt context-specific instruments to measure FL learners’ FLE.

### Gender and Emotions in Second Language Acquisition

We acknowledge that male and female L2 learners have more things in common than things that separate them. However, studies looking for differences have found that females often do better than males in L2 performance (Callaghan, 1998; Lin, 2011), which are correlated to a wide range of interacting factors such as differences in character and learning behaviors between boys and girls (Graham and Rees, 1995; Sunderland, 1998), parental influence, role models, peer pressure and image, communicative skills, reactions to different languages, contents of language lessons, career guidance, sex of the teacher, teacher expectations (Barton, 1997), and the perception that L2 learning is a female-dominant subject (Williams et al., 2002). Studies have also highlighted the fact that female learners tend to have more positive attitudes and higher motivation in learning a language than male peers which may be linked to the difference in their L2 performance (Williams et al., 2002; Kissau, 2006). These studies point to the predictive effect of gender for learners’ L2 performance.

Such predictive effect of gender has also been investigated in FL learners’ emotional experiences, especially negative emotions such as FLCA. No clear and consistent picture has emerged so far. Park and French (2013) examined gender differences in the FLCA among Korean university EFL students and found that females were more anxious. Similar results were found in L2 writing anxiety in Taiwan university EFL context (Cheng, 2002). In contrast, MacIntyre et al. (2002) investigated French FL learners in a junior high school and found that in grade 9 female students exhibited lower anxiety and higher willingness to communicate, and that boys remained constant in their overall anxiety across the three grades, while girls’ anxiety decreased from grade 8 to grade 9. A number of studies have revealed small and scattered gender effects. Matsuda and Gobel (2004) investigated general FLCA and FL reading anxieties among Japanese EFL students and found that gender had no significant predictive effect on overall general/reading anxieties and their sub-components. Similar results were found in Aida (1994), which reported no predictive effect of gender on anxiety of Japanese (L2) learners from an American University. Dewaele’s (2007) study of mature language learners in London also revealed that gender had no predictive effect on communicative anxiety levels in the first language (L1), L2, L3, and L4 in conversations with friends and in interactions with strangers. The female participants only reported more anxiety when they used their L1 (but not their L2, L3, L4, nor L5) in public speech. In short, the predictive effect of gender on language anxiety is highly variable and context-dependent.

A more consistent gender effect has been observed in recent studies into FLE and FLCA (Dewaele et al., 2016). Dewaele and MacIntyre (2014) and Dewaele et al. (2016) examined the predictive effect of gender on FLCA and FLE at different levels among a large international sample of different L1s and FLs (*N* = 1,746). Small but significant gender differences emerged for FLCA and FLE. An item-level analysis showed that the predictive effect of gender was not significant for all items on the FLCA and FLE scales. A significant predictive effect of gender was found on five of the eight items extracted from Horwitz et al. (1986) reflecting the milder expressions of anxiety, while no significant gender difference was found on the remaining three FLCA items pertaining to the more severe, paralyzing aspects of anxiety such as panic or freezing-up. In terms of FLE at item level, a significant gender difference was found for 12 items out of the 21, referring to private fun, pride, enjoyment, excitement, and interest, while no significant gender difference was found on the other nine items mostly referring to **teachers, peers, groups, and classroom environment**. The four studies consistently confirmed small but significant gender differences in overall FLE and specific items of FLE. The authors suggest that female learners may be more emotionally involved in the FL learning and may experience more emotional highs and lows than their male peers. They warn, however, against a simplistic interpretation of the findings: ‘When interpreting gender differences, we should carefully avoid thinking of the results in terms of mutually exclusive categories (as in the Mars versus Venus type of arguments), preferring instead to think of baseline differences between groups that are modified to a considerable extent by individual experiences and lead to a wide distribution of scores within each group, and considerable overlap between groups’ (p. 54).

Experiencing FLE facilitates learning because it allows the building of different types of resources and allows the exploration into FL (Dewaele and MacIntyre, 2014; Dewaele et al., 2017). Thus, the authors speculated that “females” heightened emotionality might boost the acquisition and use of the FL’ (Dewaele and Dewaele, 2017, p. 5) because having more fun in FL classes might help female learners ‘unlock … their potential faster and thus progress further than their male peers’ (Dewaele et al., 2017, p. 55). It might partially explain why female learners are generally credited as being better at FL learning than their male peers.

The varied patterns of predictive effect of gender on FLCA across different contexts are reminiscent of the patterns in FLE. Could the gender patterns of FLE found in Dewaele and MacIntyre’s (2014, 2016) international sample also be found in a specific Chinese EFL context? We find this question important for the following reasons. First, Chinese FL learners study English within a distinctive cultural, educational and social context, which plays an essential role in shaping their emotional experiences (Rao, 2006; Boiger and Mesquita, 2012). Second, most Chinese high school students are sequential bilinguals with Chinese as their L1 and English as their only FL, and the participants in this present study, unlike those in Dewaele and MacIntyre present, are seldom exposed to natural English-speaking environments. Third, Dewaele et al. (2016) claimed that different languages are perceived differently which can affect FLE and FLCA. This was confirmed in De Smet et al. (2018) who found that studying Dutch or English as an FL affected levels of FLE and FLCA of French L1 learners in Belgium. Fourth, they also found that the instruction level was significantly related to the experiences of FLE and FLCA. Given the differences of the sample in these four respects, the present study is expected to enrich the patterns of predicative effect of gender on FLE.

### Predictive Effect of Academic Discipline

Disciplinary differences have been investigated in educational research for more than 20 years (Kuteeva and Airey, 2014). Disciplines are commonly classified along the “hard-soft” and the “pure-applied” dimensions, depending on their epistemological characteristics (Becher and Trowler, 2001). On the “hard-soft” dimension, Natural Science is at the “hard” end, Social Sciences toward the middle, and Humanities at the “soft” end of the dimension (Becher and Trowler, 2001). On the “pure-applied” dimension, Natural Science is at the “pure” end, while Social Sciences and Humanities are more toward the “applied” end and more concerned with practical applications of their subject matter (Becher and Trowler, 2001). By nature, Humanities and Social Sciences generally deal with human aspects in the social world, while Natural Science disciplines with natural events. Different disciplines have different knowledge structures (Martin, 2011). Natural Science, Social Science and Humanities are characterized as having hierarchical, warring triangle, and horizontal knowledge structures (Martin, 2011; Kuteeva and Airey, 2014). Thus, people of different disciplinary backgrounds have different epistemological preferences, knowledge structures, foci, themes, methods, approaches and perspectives, which may converge to shape their distinctive academic thinking styles and behaviors.

Disciplinary effects have been found both in learning and teaching in higher education (Krause, 2014). College engineering students, for example, were reported to express “a significantly stronger preference for a logical learning and a visual learning style, whereas students with a social science background expressed significantly stronger preferences for a social learning style than for a logical learning style” (Hill et al., 2016, p. 1). It was also reported that college students from natural and social sciences had unique motivational beliefs, cognitive strategies, and domain-specific knowledge involved in self-regulating learning (Vanderstoep et al., 1996). Kuteeva and Airey (2014) found the fundamental disciplinary differences and their impact on the use of English and academic discourse in a survey at a major Swedish university and saw the impact ‘as a product of different knowledge-making practices and educational goals’ (p. 533). Disciplinary variations have also been found in college students’ trait emotional intelligence (Sánchez-Ruiz et al., 2010). Both Arts and Social Sciences students scored significantly higher than technical students in *Emotionality*, namely emotion perception of self and others, emotion expression, relationships, and trait empathy, indicating students in the former disciplines are ‘more agreeable, cooperative, and empathic’ than students in the latter (p. 54), which may help to contribute to a more positive and engaging classroom environment where student-teacher and peer interactions occur. Since a positive classroom atmosphere is a key component of FLE, it is likely that disciplinary differences may also be mirrored in FLE.

Disciplinary variations have also been uncovered among teachers, such as teaching beliefs (e.g., Nevgi et al., 2004), teaching approaches (e.g., Lindblom-Ylänne et al., 2006), teaching behaviors in classroom interaction (e.g., Murray and Renaud, 1995), and even implicit requirements for their students from different disciplines (e.g., Neumann et al., 2002). These variations on teacher side constitute an essential part of classroom dynamics and thus might influence FLE experiences of FL learners. In short, existing studies regarding discipline differences suggest FL learners’ specific discipline background, which are usually associated with variation in affective, behavioral and cognitive styles on both learning and teaching dimensions, is expected to have a predicative effect on their FLE patterns. However, empirical inquiries in this respect are scarce.

In conclusion, the existing literature suggests that gender and discipline can be sources of variation in learning and teaching in higher education and FL learners of different genders and disciplines might have different FLE experiences which can play an important role in their learning outcomes. Regrettably, empirical studies in this respect fail to include Chinese high school learners, a huge population of FL learners with their specific characteristics. To address this gap, this study aims to explore the predictive effect of gender and academic discipline on high school students’ experience of FLE in the specific Chinese EFL context.

## Research Questions

Our study is guided by the following two questions:

RQ1: What is the predictive effect of gender on the FLE?
RQ2: What is the predictive effect of students’ background academic discipline on their experience of FLE?

## Methodology

### Participants

Questionnaires in paper-and-pen format were administered in classroom situation to a sample of 1,836 second-year high school students from three schools located in central province, China. A total of 1,718 students participated in the research. Incomplete questionnaires from 118 students were discarded. The sample consists of 52% male participants (*n* = 895), and 48% female participants (*n* = 823). Participants ranged from 14 to 20 years old (*Mean* = 16.8 years old, *SD* = 0.76). A small majority (*n* = 967, 56%) were Humanities and Social Sciences students, with the remaining 751 students (44%) doing Natural Science. They attended three schools, two public ones and one private ones.

### Instrument

The Chinese Version of Foreign Language Enjoyment Scale (CFLES). The questionnaire started with a section about participants’ demographic information. This was followed by the 11-item *Chinese Version of Foreign Language Enjoyment Scale (CFLES*: Li et al., 2018), which was modified and validated based upon the *Foreign Language Enjoyment Scale (FLES*: Dewaele and MacIntyre, 2014, 2016). The *CFLES* comprises of three factors, namely *FLE-Private, FLE-Teacher* and *FLE-Atmosphere*, describing Chinese EFL learners’ FLE experiences as related to the fun, interest, and self-accomplishments in EFL learning, to the encouraging and supportive attitudes from their teachers toward them, and to the positive atmosphere or group for EFL learning (Li et al., 2018). The *CFLES* is a standard 5-point Likert scale ranging from “1 (Strongly disagree)” to “5 (Strongly agree).” The *CFLES* was confirmed as having excellent psychometric properties for its high reliability and validity (Li et al., 2018). The alphas for the global FLES, the sub-scales of *FLE-Private, FLE-Teacher*, and *FLE-Atmosphere* were 0.826, 0.792, 0.896, and 0.778, respectively, indicating high internal consistencies. The split-half reliability was found to be 0.878, indicating that the scale has high internal reliability. It also has strong construct validity, convergent validity and discriminant validity (Li et al., 2018).

### Procedure

The procedure is carried out in two-stages: obtaining consents and administering the questionnaires. In stage one, consent was firstly obtained from school presidents, headmasters, EFL teachers in each school before students’ individual consent was acquired at the start of the survey. In stage two, the questionnaires were administered to the students during self-study classes in the evening. The researcher instructed the participants in an encouraging and supportive way and they were assured in both oral and written instructions that all the data collected through the questionnaires would only be used for research purposes by the researchers, would remain confidential and that there would be no consequences for them. Paper questionnaires were used for this study because mobile phones and personal computers are not allowed in most schools at primary and secondary levels in China. Moreover, high school students are accustomed to printed questionnaires. Last but not least, the questionnaires were not completely anonymous as every student was assigned an ID in order to match the information with exam papers used in our larger related study.

### Data Analysis

We initially coped with outliers, missing values, followed by normality check and descriptive analyses. To examine the predictive effects of gender and academic discipline, independent-samples *t-test*s were conducted on the total score of the global FLE scale and each of its three sub-scales.

## Results

### Gender Differences in Foreign Language Enjoyment

The average score of FLE was 3.12 (*SD* = 0.17) and the Skewness and Kurtosis were 0.29 and 0.66 respectively, indicating that it was normally distributed, allowing for subsequent parametric analysis.

**FIGURE 1:**
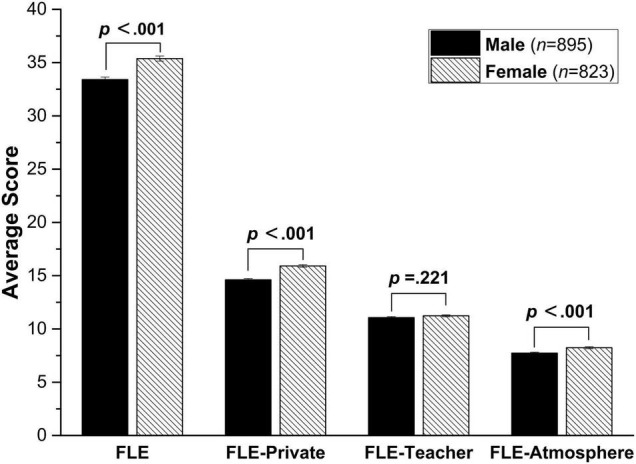
Gender differences in foreign language enjoyment (Error bars represent standard errors).

The independent-samples *t-test* showed that the female students scored significantly higher than the male students on the global FLE scale (*t* (1716) = −5.9, *p* < 0.0001, *d* = −0.14) and the two subscales *FLE-Private (t* (1716) = −7.2, *p* < 0.0001, *d* = 0.69) and *FLE-Atmosphere (t* (1716) = −3.8, *p* < 0.0001, *d* = 2.56) (See Figure 1). However, no significant gender effect emerged on *FLE-Teacher (t* (1716) = −1.2, *p* = 0.221, *d* = 0.09).

### Disciplinary Differences in Foreign Language Enjoyment

**FIGURE 2:**
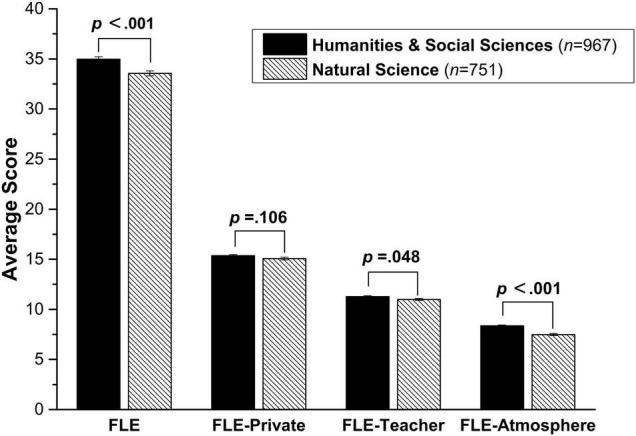
Disciplinary differences in foreign language enjoyment (Error bars represent standard errors).

To examine the predictive effect of discipline, an independent-samples *t-test* was conducted on the overall global FLE scale and each of its three sub-scales. The results showed that Humanities and Social Sciences students scored significantly higher than the Natural Science students on the global FLE scale (*t* (1716) = 4.2, *p* < 0.0001, *d* = 0.21) and on two subscales: *FLE-Teacher* (*t* (1716) = 2.0, *p* < 0.048, *d* = 0.10) and *FLE-Atmosphere* (*t* (1666.5) = −3.8, *p* < 0.0001, *d* = 0.04) (See Figure 2). However, there was no significant difference between Humanities & Social Sciences students and Natural Science students on *FLE-Private* (*t* (1716) = 1.6, *p* = 0.106, *d* = 0.37).

## Discussion

### Predictive Effect of Gender

The present study revealed significant gender differences in overall FLE, and its three dimensions: *FLE-Private, FLE-Teacher*, and *FLE-Atmosphere.* Female participants scored significantly higher than their male counterparts on the overall FLE scale as well as on the sub-scales of *FLE-Private* and *FLE-Atmosphere*. However, no significant gender difference emerged for the *FLE-Teacher* dimension. Broadly similar gender patterns were found in both FLE and FLCA in Dewaele and MacIntyre (2014) and Dewaele et al. (2016) which shared the same international dataset. A comparison of the present findings with those uncovered previously (see Table 1) reveals similar predictive effects of gender on items referring to private enjoyment, interest, pride, and fun, while no predictive effect of gender was found for items referring to teachers. Similarly, Williams et al. (2002) found that female participants showed higher desire, motivation and liking in FL learning, but female and male participants had similar attitudes toward teachers. The predictive effect of gender on *FLE-Atmosphere* in the present study echoes with the observation by Beer and Darkenwald (1989) that ‘women perceive a greater degree of involvement in the classroom than did men’ (p. 33).

**TABLE 1:** Comparison of Dewaele et al. (2016) and the present study in the effects of gender on FLE at dimension and item levels.

	Items and dimensions in the present study	*p* value	
		Present study	Dewaele et al. (2016)
	**Dimension 1: *FLE-Private***		
1.	I don’t get bored.	0.001	0.033
2.	I enjoy it.		0.006
3.	I’ve learnt interesting things.		0.002
4.	In class, I feel proud of my accomplishments.		0.001
6.	It’s fun.		0.001
	**Dimension 2: *FLE-Atmosphere***		
5.	It’s a positive environment.	0.001	0.045
10.	There is a good atmosphere.		0.518
11.	We form a tight group.		0.127
	Dimension 3: *FLE-Teacher*		
7.	The teacher is encouraging.	0.221	0.600
8.	The teacher is friendly.		0.533
9.	The teacher is supportive.		0.903

The absence of gender differences for *FLE-Teacher* was also reflected in the qualitative study reported in Li et al. (2018). In the descriptions of enjoyable episodes in FL learning, three quarters of the 49 participants (26 males, 23 females) mentioned their teachers, praising them for showing their attention and for encouraging students. Similar observations were reported in Dewaele and MacIntyre (2014) but teachers were mentioned in only 20% of comments.

The strong link between teacher and FLE in the present study points to a unique feature of Chinese culture, namely a ‘high acceptance of power and authority’ (Rao, 2006, p. 494) and hence the dominant role of teachers as source of FLE. In Chinese FL learning, ‘students are more accustomed to teacher-centered classroom activities’ (p. 496), and the teacher sets the emotional tone in FL classes as they are in complete control. All students are aware of teachers’ authority and power, which makes them vulnerable to teachers’ feedback, attitude and pedagogical practices. FL enjoyment, pride and interest rest in the teachers’ hands. It is possible that this cancels out gender differences in *FLE-Teacher*. This absence of gender difference in *FLE-Teacher* could also be explained by the fact that Chinese EFL teachers usually have to teach a large class with more than 40 students. While teaching so many students in a class, it is hardly possible to attend closely to students’ individual cognitive and emotional needs that might divide the male and the female, which will probably contribute to a shared experience of FLE-teacher among the girls and the boys. Last but not least, in China, equality between man and woman is deeply rooted and in educational context of China, the teachers don’t seem to discriminate between boys and girls in their interaction with them.

In the present study, girls reported experiencing more overall FLE in FL classes than boys, supporting the findings in Gentry et al. (2002). The result is also consistent with the commonly-held stereotype that females tend to be more emotional than males (Fischer and Manstead, 2000; Dewaele and MacIntyre, 2014). Gender differences tend to occur more in emotion-related display in different contexts and could be explained based upon the Social Role Theory (Eagly, 1987). According to the theory, gender differences in emotional experiences develop as a consequence of awareness of the contents of masculinity and femininity, which are partly determined by the biological origin of the division of labor between the sexes and associated sex-role ideology. That is, emotions could be interpreted as an extension of gender roles and gender ideology (Fischer and Manstead, 2000). There is evidence from countries all over the world that the primary social division of labor for females is to be caretakers of their children. Therefore, emotionally, the traditional gender role for females is to be relationally-oriented caregivers (Chaplin and Aldao, 2013). That is why girls are often characterized as being more emotional than males, expressing more emotions than boys (Dewaele and MacIntyre, 2014), particularly pro-social emotions (e.g., enjoyment, happiness, joy, enthusiasm, and empathy) and internalizing negative emotions implying vulnerability or powerlessness (e.g., anxiety, shame, guilt, fear and sadness) (Fischer and Manstead, 2000; Brody and Hall, 2008).

It seems that specifically in the FL learning context, the gender difference in overall FLE mirrors the gender difference in FL performance and motivation. Based upon findings in the present and previous related studies, we suggest that FL performance and FLE can boost each other and that there may be a mutual influence between gender differences in FL achievement and gender differences in FLE.

First, females enjoy FL learning more compared with males (Dewaele et al., 2016), which in turn can lead to their better L2 performance compared with males. Considering the facilitative role of enjoyment in learning proposed in the Control-value Theory and the Broaden-and-Build Theory, gender differences in FLE may be linked to cognition, behavior, and ultimate success in language learning (Goetz et al., 2008). Besides, enjoyment of learning feeds into intrinsic and extrinsic motivation as well as academic effort (Pekrun and Linnenbrink-Garcia, 2014, p. 47). Graham and Rees (1995, p. 18) also found that ‘enjoyment is a strong motivating force’, and that girls consistently experience more enjoyment and interest in class than the boys across grade levels, which allow them to be more motivated in learning (Gentry et al., 2002). The link between gender, enjoyment and motivation has been established before: Kissau (2006) claimed that females typically outperform males in L2 acquisition because of their general higher levels of L2 motivation.

Second, female learners generally outperform their male peers on FL standardized tests (Williams et al., 2002; Park and French, 2013), which in turn can contribute to boosting their FLE. Male learners, on the other hand, might suffer not just from an absence of any boost but also by the disappointment of not doing very well. This assumption was empirically supported in Goetz et al. (2008). They found stereotypical gender differences in achievement and academic enjoyment in two domains of language class and mathematics class. Compared to male students, female students had higher grades and more enjoyment in German language course while lower grades and less enjoyment in mathematics course. And for the within-domain relationships, they revealed positive and medium size path coefficients from achievement to enjoyment, indicating that gender differences in language achievement may contribute to the gender difference in FLE.

### Predictive Effect of Academic Discipline

As shown in Figure 2, Humanities and Social Sciences students scored significantly higher than Natural Science students on the overall *FLE*, *FLE-Teacher* and *FLE-Atmosphere*, echoing the findings in Emotionality at university level (Sánchez-Ruiz et al., 2010). However, no disciplinary predictive effect was found on *FLE-Private.* A number of possible causes could explain this disciplinary effect. First, the foci of different disciplines may have an indirect predictive effect on emotional experience during FL learning process. Compared to the Natural Science discipline, Humanities and Social Sciences are more about different aspects of human interactions and strengthening students’ humanistic qualities. And in Humanities and Social Sciences, ‘a people-oriented approach is dominant and one-to-one interactions are often required’ (Sánchez-Ruiz et al., 2010, p. 55), and thus traits such as emotion perception, expression and empathy are more important (p. 55). Humanities and Social Sciences students are therefore more exposed to emotion-related issues in human interactions in their study, which may facilitate directly or indirectly the enhancement of their meta-emotion ability and awareness of social intelligence. Besides, the subjects in Humanities and Social Sciences are more humanistic and more open to discussion, which will also contribute to the establishment of a more open and emotionally activated classroom climate where both teachers and students are more emotionally involved. This is consistent with the common practice reported by several EFL teachers that Humanities and Social Sciences classes are more cooperative, active and positively engaged, while Natural Science classes are typically quieter, even cold or aloof, and they receive few responses from the students. Naturally, a more positive classroom environment is more likely to boost FLE, especially *FLE-Atmosphere* and *FLE-Teacher*.

Furthermore, the adjacency between English and other subjects (i.e., Politics, History and Geography) within the same domain of Humanities and Social Sciences may facilitate FL learning and enhance FL performance, thus indirectly helping to boost FLE as discussed previously. Situated within the same domain, English and other subjects have shared values in providing students a place where they can find out what makes human, how they can negotiate social identities, explore and engage in different cultures and experience diverse ways of being, doing and seeing, which is quite different from what Natural Sciences can offer. The resemblance, overlapping, and connectedness in objects and knowledge structure may facilitate EFL learning. Just as an experienced EFL teacher and headmaster in our study said, compared to Natural Science disciplines, Politics, History and Geography involves more extensive reading, which can help to broaden students’ horizon and knowledge scope, and are more about language, language reading, comprehension and communication, and culture in society. Thus, this cross-disciplinary learning within Humanities and Social Sciences provide more language-related training, especially in reading comprehension and text analysis, which will probably facilitate both EFL learning and teaching. And this advantage in FL learning may further contribute to differences in overall FLE experience.

Although students in different disciplines have the same curriculum for English, the same amount of teaching hours, and even the same English teachers, they may have different perceptions of English among all six subjects they have to learn, which may influence their academic investment in English study. In general, Natural Science students have more homework, including challenging exercises in Physics, Chemistry or Biology. These students consider the three subjects and Mathematics as more difficult than English and Chinese, and they invest more time in working out a challenging problem in Mathematics or Physics instead of English. However, for students of Humanities and Social Sciences, Chinese, Politics, History and Geography involve more repetitive work of understanding and memorizing, and are less challenging compared to Mathematics and English. Thus, they prioritize and invest more in Mathematics and English. According to Dewaele and MacIntyre (2016, p. 217) ‘enjoyment follows personal investment and requires having a stake in an outcome that matters to the person,’ the difference of investment on EFL learning may also have potential links with the difference of overall FLE experience.

The absence of a disciplinary predictive effect in *FLE-Private* indicates that students from both disciplines experience similar levels of private enjoyment coalescing around interesting things, fun, novelty and pride of accomplishments they have come across during FL exploration. This also indicates that the disciplinary differences in FLE are more related to external or environmental aspects instead of private aspects.

We are aware of three main limitations in the present study. First, although the sample is large, all the participants were at the same instructional level and from the same province of China, thus not necessarily representing the whole situation in China. Second, the scale is based on self-reports, which means that a social desirability bias cannot be excluded. In other words, participants may have exaggerated their levels of FLE to please the researchers. Third, mixed sequential studies can be carried out in the future to triangulate the findings of this present study.

## Conclusion

This study based on a large sample of Chinese EFL students has revealed that both gender and discipline are sources of inter-individual variation in FLE. First, female students reported more overall *FLE, FLE-Private* and *FLE-Atmosphere* than male students. In this respect, gender differences are very similar to those uncovered in different parts of the world (Dewaele and MacIntyre, 2014; Dewaele et al., 2016). In contrast, no significant gender difference emerged for *FLE-Teacher.* This might be linked to the prominent role of teachers in Chinese EFL classes and the Chinese cultural value of respect for authority. Students’ comments on enjoyable episodes in the EFL class were linked much more frequently to the teacher than in Dewaele and MacIntyre (2014).

Second, students of Humanities and Social Sciences scored significantly higher than Natural Science students in overall *FLE, FLE-Teacher* and *FLE-Atmosphere*, but no significant difference was found in their *FLE-Private.* This finding echoes previous research on the predictive effect of discipline around the world. It suggests that disciplinary differences in FLE are more related to social context rather than private experience.

The findings have some pedagogical implications. First, EFL teachers should hold a holistic view on their students, paying attention not only to differences in FL performance but also to students’ emotional states and the emotional temperature of the classroom (Moskowitz and Dewaele, 2019; Li et al., 2021). In other words, a holistic view, which echoes the key tenet of Positive Psychology as well as the humanistic English teaching (Arnold, 1998), might be the key to boosting FLE. Another implication is that FL teachers should not just focus on negative emotions (Li, 2020). EFL teachers might consider and devise ways to specifically boost male students’ FLE. Besides, language teachers should be aware of the disciplinary differences in overall FLE and find novel ways to engage students from a hard science background. Positive Psychology interventions might help, with an emphasis on boosting interest, enjoyment, and creating a positive classroom environment (Li and Xu, 2019).

In sum, successful FL learning is dependent on the harmonious “dance” between teachers and students, between peers, or even between students and their FL selves (Gregersen and MacIntyre, 2017). It is essential for teachers and students to develop harmonious relationships in order to boost motivation and academic success.

Funding Project: The present article is part of the research project “Non–verbal emotional interaction and the effectiveness of foreign language classroom teaching”, supported by the National Social Science Foundation of China (15BYY082).

## Data Availability Statement

The raw data supporting the conclusions of this article will be made available by the authors, without undue reservation.

## Author Contributions

JH included the selection of topic, and the collection and analysis of the data as well as the draft writing and revision. GJ included suggestions on research focus, data analysis, and draft revisions. Both authors contributed to the article and approved the submitted version.

## Conflict of Interest

The authors declare that the research was conducted in the absence of any commercial or financial relationships that could be construed as a potential conflict of interest.

## Publisher’s Note

All claims expressed in this article are solely those of the authors and do not necessarily represent those of their affiliated organizations, or those of the publisher, the editors and the reviewers. Any product that may be evaluated in this article, or claim that may be made by its manufacturer, is not guaranteed or endorsed by the publisher.
